# Impact of the Covid-19 Pandemic on Birth Rates in 2020: The Case of Colombia

**DOI:** 10.1055/s-0041-1731380

**Published:** 2021-07-27

**Authors:** Vicky Margarita Montaño Mendoza, Paula Andrea Velilla, Sergio Tamayo Hussein, Walter Cardona Maya

**Affiliations:** 1 Reproduction Group, Departament of Microbiology and Parasitology, Facultad de Medicina, Universidad de Antioquia, Antioquia, Colombia; 2 Immunovirology Group, Departament of Microbiology and Parasitology, Facultad de Medicina, Universidad de Antioquia, Antioquia, Colombia; 3 Instituto de fertilidad Humana - InSer, Medellín, Colombia

The global emergency arising from the rapid spread of the severe acute respiratory syndrome coronavirus 2 (SARS-COV-2) virus, the etiological agent responsible for coronavirus disease 2019 (Covid-19), has resulted in a significant loss of more than 2.5 million human lives across the world until now. On March 12, 2020, Colombia's authorities declared a state of emergency due to Covid-19, and, by March 25, 2021, have reported 2,359,942 cases and 62,519 deaths attributed to the infection ( https://www.ins.gov.co ). Following the recommendation of the World Health Organization (WHO), just a year ago, Colombia's national government adopted mandatory preventive isolation to reduce new cases of Covid-19. However, despite implementing an early lockdown and having a 97,78% health coverage by the end of 2020, there is still great concern about the quality of care to respond effectively to the needs of infected patients, considering the 29% of accumulated excess of total mortality in 2020, compared with the national historical average. 1

Besides the direct impact of the novel disease on individual health, the Covid-19 crisis has had significant consequences on the economic growth of the affected populations. Although the non-pharmaceutical interventions applied in most countries, including Colombia, to control the viral transmission and prevent the collapse of health care services, such as lockdowns, isolation, social distancing, and quarantine measures, have shown to be beneficial, they prompted a decline in productivity with a subsequent economic downturn, representing a significant challenge in some vulnerable countries with less developed health systems and lacking the financial ability to respond to the pandemic. 2

Economic and social changes triggered by the widespread community transmission of the SARS-CoV-2 infection also negatively affect the population who wants to get pregnant, influencing fertility and birth rates.

The changes imposed by the pandemic not only affected the prenatal control program in Colombia, but also altered the mental state, intimacy, relationship dynamics, and reproductive desires in various ways worldwide. For instance, many Italian couples trying to conceive abandoned their intention to do so, precisely due to concerns of further economic difficulties and the repercussions on gestation. 3 Moreover, it has been reported that physiological changes during pregnancy may impact on the outcomes of mothers and newborns with Covid-19, 4 increasing the perception of pregnancy as a threat on the part of couples attempting conception. Collected statistics of existing research on sexual health during the pandemic closely relate to such remarks. Studies 5 report a significant decrease in sexual activity, probably due to the major changes at the beginning of the epidemic plus the perceived high risk of sexual practices. Altogether, current evidence thus raises awareness about the impairment in sexual and reproductive health and its forthcoming impact on the demographic composition of the affected nations.

An analysis using information from the Colombian National Administrative Department of Statistics (DANE, https://www.dane.gov.co/ ) regarding reports on births in Colombia from 2014 to 2020 was recorded for this letter. The data from 2014 to 2019 was consolidated and compared with the data reported for 2020. Birth records for 2020 (preliminary report) were also compared with the average of the last 6 years (2014 to 2019) by yearly range ( Fig. 1 ).

**Figure:**
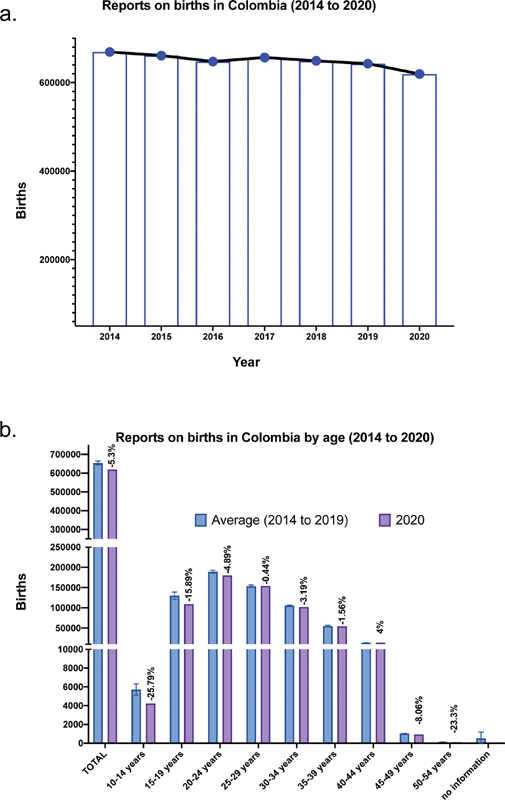
Fig. 1 The total (a) and range per age (b) of birth data for 2020 with respect to the data reported for the last 6 years (2014 to 2019).

The number of live births in Colombia during the last 6 years (2014 to 2019) is broadly stable ( Fig. 1a ). However, there is a notable reduction of 5.32% in total births in 2020 compared with the mean national value for the past 6 years. When comparing the national average of the previous 6 years with 2020 ranges per age ( Fig. 1b ), we also observed a decline in births in most age groups, with the highest decrease in women aged 10 to 14.

Although throughout the first 3 quarters of 2020 the number of births remained stable and comparable to that of previous years, it was in the last quarter of 2020 that births started to decline progressively, not redressing the rise in the mortality rate and the fatality rate of Covid-19. A similar situation occurs in France, Spain, and the United States, where there are important drops in births, contrary to the expected baby boom at this pandemic stage, based on prior epidemiological predictions.

The decrease in total births in Colombia in 2020, compared with average birth rates, has several implications. First, the social measures aimed to flatten the Covid-19 curve and lessen the economic impact are probably the main cause behind the negative effect on the fertility rate, both in high- and lower-to-middle-income economies. Notably, the economic crisis and employment uncertainty derived from policies against the spread of the virus might be the most significant circumstances accountable for such a decline, given their known influence in the reproductive decision-making as, in general, falling income has been associated to the postponement and abandonment of planned births, which may not be reversed regardless of improvements in economic indexes. 6 7 After that, the fall in fertility and birth rates has implications on the demographic structure, and ultimately, on the age distribution of the population, having an aggregate effect on economic affluence. 8

The drop in the number of total births in 2020 in Colombia, will probably be followed by a gradual and modest increase in fertility rates in subsequent years, once the social and economic turbulence is over, in a pattern similar to how most epidemics affect demographyics. 9 As for now, even when vaccine efforts are being successful, the economic fallout will plausibly continue; hence, it is unlikely that the declining fertility trends alleviate in the short term, notwithstanding an apparent economic recovery. Furthermore, it is imperative to intervene on the main determinants regarding reproductive decision-making: are age, gender, schooling, and socioeconomic status. However, the large decline in newborns of women aged 10 to 14 years should be maintained through prioritizing investments in programs to educate men and women on sexual and reproductive health, encouraging planned births only among couples with favorable conditions to be parents. Lastly, this warrants epidemiological sociodemographic-economic models in line with the currently available data, aiming to design up-to-date strategies focused on the control of virus outbreaks, the protection of economic prosperity against possible upcoming public health crises, and an increase in birth rates.

## References

[1] Ministerio de Salud Protección Social de Colombia Vigilancia demográfica de la mortalidad por COVID-19 en Colombia 2020 [Internet]. Bogotá: Dirección de Epidemiología y Demografía 2021[cited 2021 Feb 12]. Available from:https://www.minsalud.gov.co/sites/rid/Lists/BibliotecaDigital/RIDE/VS/ED/VSP/vigilancia-demografica-mortalidad-covid-19-colombia2020.pdf

[2] Nicola M Alsafi Z Sohrabi C Kerwan A Al-Jabir A Iosifidis C The socio-economic implications of the coronavirus pandemic (COVID-19): A review Int J Surg 2020 78 185 193 10.1016/j.ijsu.2020.04.018 32305533 PMC7162753

[3] Micelli E Cito G Cocci A Polloni G Russo G I Minervini A Desire for parenthood at the time of COVID-19 pandemic: an insight into the Italian situation J Psychosom Obstet Gynaecol 2020 41 03 183 190 10.1080/0167482X.2020.1759545 32379999

[4] Di Toro F Gjoka M Di Lorenzo G De Santo D De Seta F Maso G Impact of COVID-19 on maternal and neonatal outcomes: a systematic review and meta-analysis Clin Microbiol Infect 2021 27 01 36 46 10.1016/j.cmi.2020.10.007 33148440 PMC7605748

[5] Delcea C Chirila V I Săuchea A M Effects of COVID-19 on sexual life–a meta-analysis Sexologies 2021 30 01 e49 e54 10.1016/j.sexol.2020.12.001

[6] Adsera A Menendez A Fertility changes in Latin America in periods of economic uncertainty Popul Stud (Camb) 2011 65 01 37 56 10.1080/00324728.2010.530291 21213181 PMC3616445

[7] Hanappi D Ryser V A Bernardi L Le Goff J M Changes in employment uncertainty and the fertility intention–realization link: an analysis based on the Swiss household panel Eur J Popul 2017 33 03 381 407 10.1007/s10680-016-9408-y 28725099 PMC5493711

[8] Prettner K Bloom D E Strulik H Declining fertility and economic well-being: do education and health ride to the rescue? Labour Econ 2013 22 70 79 10.1016/j.labeco.2012.07.001 26388677 PMC4572580

[9] Mamelund S E Fertility fluctuations in times of war and pandemic influenza J Infect Dis 2012 206 01 140 141, author reply 141–143 10.1093/infdis/jis315 22535995

